# 8-Hy­droxy-2-methyl­quinolinium tetra­chlorido(pyrazine-2-carboxyl­ato-κ^2^ *N* ^1^,*O* ^2^)stannate(IV) methanol monosolvate

**DOI:** 10.1107/S1600536811032296

**Published:** 2011-08-17

**Authors:** Marzieh Vafaee, Ezzatollah Najafi, Mostafa M. Amini, Seik Weng Ng

**Affiliations:** a Department of Chemistry, General Campus, Shahid Beheshti University, Tehran 1983963113, Iran; b Department of Chemistry, University of Malaya, 50603 Kuala Lumpur, Malaysia; c Chemistry Department, Faculty of Science, King Abdulaziz University, PO Box 80203 Jeddah, Saudi Arabia

## Abstract

In the title solvated salt, (C_10_H_10_NO)[SnCl_4_(C_5_H_3_N_2_O_2_)]·CH_3_OH, the Sn^IV^ atom is chelated by the *N*,*O*-bidentate pyrazine-2-carboxyl­ate ligand and four chloride ions, and shows a distorted octa­hedral SnNOCl_4_ coordination at the metal atom. The 8-hy­droxy-2-methyl­quinolinium cation and the anion are linked to the methanol mol­ecules by O—H⋯O, O—H⋯N and N—H⋯O hydrogen bonds, generating a linear chain running along [1
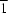
0]. There are two independent ion pairs and solvent mol­ecules in the asymmetric unit. The crystal studied was a non-merohedral twin with a 41.8 (1)% twin component.

## Related literature

For another ammonium tetra­chlorido(pyrazine-2-carboxyl­ato)stannate(IV), see: Najafi *et al.* (2011 ▶).
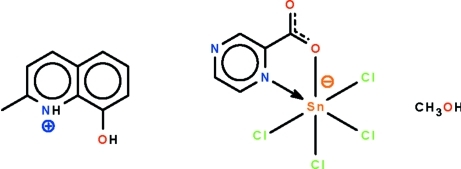


## Experimental

### 

#### Crystal data

(C_10_H_10_NO)[SnCl_4_(C_5_H_3_N_2_O_2_)]·CH_4_O
*M* *_r_* = 575.82
Triclinic, 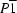

*a* = 6.8392 (2) Å
*b* = 16.9759 (8) Å
*c* = 17.6637 (10) Å
α = 90.337 (4)°
β = 94.429 (4)°
γ = 92.232 (3)°
*V* = 2043.03 (16) Å^3^
*Z* = 4
Mo *K*α radiation
μ = 1.80 mm^−1^
*T* = 100 K
0.25 × 0.25 × 0.10 mm

#### Data collection

Agilent SuperNova Dual diffractometer with an Atlas detector
Absorption correction: multi-scan (*CrysAlis PRO*; Agilent, 2010 ▶) *T*_min_ = 0.661, *T*_max_ = 0.840
11717 measured reflections
11717 independent reflections
10323 reflections with *I* > 2σ(*I*)
*R*_int_ = 0.074

#### Refinement

*R*[*F* ^2^ > 2σ(*F* ^2^)] = 0.038
*wR*(*F* ^2^) = 0.116
*S* = 1.08
11717 reflections
514 parameters
H-atom parameters constrained
Δρ_max_ = 1.47 e Å^−3^
Δρ_min_ = −1.44 e Å^−3^

### 

Data collection: *CrysAlis PRO* (Agilent, 2010 ▶); cell refinement: *CrysAlis PRO*; data reduction: *CrysAlis PRO*; program(s) used to solve structure: *SHELXS97* (Sheldrick, 2008 ▶); program(s) used to refine structure: *SHELXL97* (Sheldrick, 2008 ▶); molecular graphics: *X-SEED* (Barbour, 2001 ▶); software used to prepare material for publication: *publCIF* (Westrip, 2010 ▶).

## Supplementary Material

Crystal structure: contains datablock(s) global, I. DOI: 10.1107/S1600536811032296/zs2137sup1.cif

Structure factors: contains datablock(s) I. DOI: 10.1107/S1600536811032296/zs2137Isup2.hkl

Additional supplementary materials:  crystallographic information; 3D view; checkCIF report

## Figures and Tables

**Table 1:** Hydrogen-bond geometry (Å, °)

*D*—H⋯*A*	*D*—H	H⋯*A*	*D*⋯*A*	*D*—H⋯*A*
O5—H5o⋯O7	0.84	1.84	2.661 (5)	167
O7—H7o⋯N2	0.84	2.05	2.871 (5)	165
O6—H6o⋯O8	0.84	1.82	2.649 (5)	171
O8—H8o⋯N4	0.84	2.10	2.924 (5)	168
N5—H5n⋯O4	0.88	1.88	2.731 (5)	163
N6—H6n⋯O2^i^	0.88	1.98	2.838 (5)	164

Symmetry code: (i) 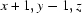.

**Fig. 1.**
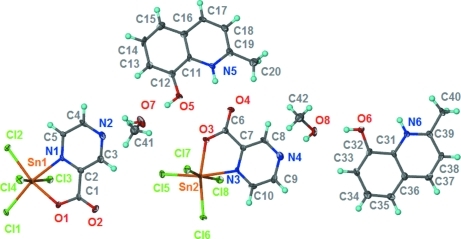
Thermal ellipsoid plot (Barbour, 2001) of (C10H10NO)[SnCl4(C5H3N2O2)].CH3OH, at the 70% probability level. Hydrogen atoms are drawn as spheres of arbitrary radius.

## supplementary crystallographic information

### Comment

In a recent study, we reacted stannic chloride with pyrazine-2-carboxylic acid in methanol, the reaction yielding the tetrachlorido(pyrazine-2-carbxoylato)stannate anion, whose charge was balanced by a 3-methoxycarbonyl-1-methylpyrazinium cation (Najafi *et al.*, 2011). The cation was derived from the original carboxylic acid which was simultaneously esterified and *N*-methylated. A similar synthesis but with 2-methyl-8-hydroxyquinoline in methanol medium yielded the solvated title salt (Scheme I, Fig. 1). The less sterically crowded carboxylate ligand engages in chelation, and the more crowded quinoline ligand is protonated. The Sn^IV^ atom shows distorted octahedral SnNOCl_4_ coordination. The cation and anion are linked to the methanol molecules by O–H···O and N–H···O hydrogen bonds (Table 1). There are two independent ion-pairs and solvent molecules. The crystal studied is a non-merohedral twin with a 41.8 (1)% twin component.

### Experimental

Stannic chloride pentahydrate (0.35 g, 1 mmol), pyrazine-2-carboxylic acid (0.13 g, 1 mmol) and 2-methyl-8-hydroxyquinoline (0.16 g, 1 mmol) were loaded into a convection tube and the tube was filled with dry methanol and kept at 333 K. Colorless crystals were collected from the side arm after several days.

### Refinement

Carbon-, nitrogen- and oxygen-bound H-atoms were placed in calculated positions [C—H = 0.95 to 0.98 Å, N—H = 0.88 Å, O—H = 0.84 Å] and *U*_iso_(H) = 1.2 to 1.5*U*_eq_(C,*N*,*O*)] and were included in the refinement in the riding model approximation. The final difference Fourier map had a peak in the vicinity of Sn2 and a hole in the vicinity of Sn1. The crystal studied is a non-merohedral twin with a minor component of 41.8 (1)%.

### Crystal data

**Table:**

(C_10_H_10_NO)[SnCl_4_(C_5_H_3_N_2_O_2_)]·CH_4_O	*Z* = 4
*M**_r_* = 575.82	*F*(000) = 1136
Triclinic, *P*1	*D*_x_ = 1.872 Mg m^−^^3^
Hall symbol: -P 1	Mo *K*α radiation, λ = 0.71073 Å
*a* = 6.8392 (2) Å	Cell parameters from 4896 reflections
*b* = 16.9759 (8) Å	θ = 2.3–27.5°
*c* = 17.6637 (10) Å	µ = 1.80 mm^−^^1^
α = 90.337 (4)°	*T* = 100 K
β = 94.429 (4)°	Prism, colorless
γ = 92.232 (3)°	0.25 × 0.25 × 0.10 mm
*V* = 2043.03 (16) Å^3^	

### Data collection

**Table:**

Agilent SuperNova Dual diffractometer with an Atlas detector	11717 independent reflections
Radiation source: SuperNova (Mo) X-ray Source	10323 reflections with *I* > 2σ(*I*)
mirror	*R*_int_ = 0.074
Detector resolution: 10.4041 pixels mm^-1^	θ_max_ = 27.5°, θ_min_ = 2.3°
ω scans	*h* = −8→8
Absorption correction: multi-scan (*CrysAlis PRO*; Agilent, 2010)	*k* = −21→21
*T*_min_ = 0.661, *T*_max_ = 0.840	*l* = −22→22
11717 measured reflections	

### Refinement

**Table:**

Refinement on *F*^2^	Primary atom site location: structure-invariant direct methods
Least-squares matrix: full	Secondary atom site location: difference Fourier map
*R*[*F*^2^ > 2σ(*F*^2^)] = 0.038	Hydrogen site location: inferred from neighbouring sites
*wR*(*F*^2^) = 0.116	H-atom parameters constrained
*S* = 1.08	*w* = 1/[σ^2^(*F*_o_^2^) + (0.0698*P*)^2^] where *P* = (*F*_o_^2^ + 2*F*_c_^2^)/3
11717 reflections	(Δ/σ)_max_ = 0.001
514 parameters	Δρ_max_ = 1.47 e Å^−^^3^
0 restraints	Δρ_min_ = −1.44 e Å^−^^3^

### Fractional atomic coordinates and isotropic or equivalent isotropic displacement parameters (Å^2^)

**Table:**

	*x*	*y*	*z*	*U*_iso_*/*U*_eq_	
Sn1	0.61697 (4)	1.596168 (18)	0.654851 (18)	0.00792 (8)	
Sn2	1.08405 (4)	1.091896 (18)	0.846561 (18)	0.00807 (9)	
Cl1	0.45746 (16)	1.69944 (7)	0.71191 (7)	0.0121 (2)	
Cl2	0.57579 (16)	1.63204 (7)	0.52408 (6)	0.0136 (2)	
Cl3	0.32606 (16)	1.51100 (7)	0.64196 (7)	0.0123 (2)	
Cl4	0.93371 (16)	1.65961 (7)	0.66605 (7)	0.0158 (3)	
Cl5	0.91591 (17)	1.19680 (7)	0.78769 (7)	0.0137 (2)	
Cl6	1.07721 (16)	1.12849 (7)	0.97661 (6)	0.0127 (2)	
Cl7	1.40092 (16)	1.15454 (7)	0.84076 (7)	0.0140 (2)	
Cl8	0.79318 (16)	1.00802 (7)	0.85129 (7)	0.0133 (2)	
O1	0.6790 (4)	1.54266 (19)	0.75974 (18)	0.0112 (7)	
O2	0.8132 (5)	1.4405 (2)	0.82093 (19)	0.0158 (7)	
O3	1.1240 (5)	1.03584 (19)	0.74318 (18)	0.0118 (7)	
O4	1.2544 (5)	0.9339 (2)	0.68656 (19)	0.0171 (8)	
O5	1.2786 (5)	1.0859 (2)	0.59595 (19)	0.0154 (7)	
H5O	1.2615	1.1322	0.6103	0.023*	
O6	1.8052 (5)	0.5901 (2)	0.92417 (19)	0.0147 (7)	
H6O	1.8119	0.6375	0.9110	0.022*	
O7	1.2839 (5)	1.2368 (2)	0.6382 (2)	0.0228 (9)	
H7O	1.1952	1.2673	0.6235	0.034*	
O8	1.7831 (5)	0.7393 (2)	0.8827 (2)	0.0188 (8)	
H8O	1.6821	0.7647	0.8882	0.028*	
N1	0.7781 (5)	1.4869 (2)	0.6269 (2)	0.0089 (8)	
N2	0.9857 (6)	1.3506 (2)	0.6162 (2)	0.0134 (9)	
N3	1.2494 (5)	0.9823 (2)	0.8807 (2)	0.0081 (8)	
N4	1.4662 (6)	0.8481 (2)	0.8964 (2)	0.0138 (9)	
N5	1.2367 (5)	0.9428 (2)	0.5319 (2)	0.0094 (8)	
H5N	1.2372	0.9503	0.5812	0.011*	
N6	1.7531 (5)	0.4442 (2)	0.9781 (2)	0.0094 (8)	
H6N	1.7733	0.4534	0.9303	0.011*	
C1	0.7736 (6)	1.4772 (3)	0.7640 (3)	0.0092 (9)	
C2	0.8332 (6)	1.4461 (3)	0.6889 (3)	0.0099 (9)	
C3	0.9371 (7)	1.3782 (3)	0.6833 (3)	0.0136 (10)	
H3	0.9750	1.3504	0.7282	0.016*	
C4	0.9310 (6)	1.3919 (3)	0.5549 (3)	0.0133 (10)	
H4	0.9660	1.3745	0.5067	0.016*	
C5	0.8231 (6)	1.4605 (3)	0.5591 (3)	0.0098 (9)	
H5	0.7823	1.4877	0.5142	0.012*	
C6	1.2204 (7)	0.9719 (3)	0.7426 (3)	0.0115 (9)	
C7	1.2951 (6)	0.9421 (3)	0.8197 (3)	0.0095 (9)	
C8	1.4038 (7)	0.8751 (3)	0.8276 (3)	0.0135 (10)	
H8	1.4351	0.8476	0.7834	0.016*	
C9	1.4166 (6)	0.8884 (3)	0.9566 (3)	0.0130 (10)	
H9	1.4571	0.8707	1.0060	0.016*	
C10	1.3069 (7)	0.9558 (3)	0.9490 (3)	0.0134 (10)	
H10	1.2731	0.9829	0.9931	0.016*	
C11	1.2567 (7)	1.0078 (3)	0.4856 (3)	0.0099 (9)	
C12	1.2771 (6)	1.0836 (3)	0.5197 (3)	0.0112 (9)	
C13	1.2956 (7)	1.1475 (3)	0.4735 (3)	0.0132 (10)	
H13	1.3116	1.1988	0.4953	0.016*	
C14	1.2913 (7)	1.1381 (3)	0.3940 (3)	0.0166 (11)	
H14	1.3013	1.1833	0.3628	0.020*	
C15	1.2730 (7)	1.0648 (3)	0.3611 (3)	0.0177 (11)	
H15	1.2730	1.0590	0.3076	0.021*	
C16	1.2541 (6)	0.9978 (3)	0.4072 (3)	0.0123 (10)	
C17	1.2341 (7)	0.9196 (3)	0.3781 (3)	0.0143 (10)	
H17	1.2339	0.9104	0.3249	0.017*	
C18	1.2155 (7)	0.8578 (3)	0.4256 (3)	0.0143 (10)	
H18	1.2014	0.8059	0.4051	0.017*	
C19	1.2165 (6)	0.8689 (3)	0.5043 (3)	0.0127 (10)	
C20	1.1981 (7)	0.8021 (3)	0.5576 (3)	0.0192 (11)	
H20A	1.2089	0.8223	0.6099	0.029*	
H20B	1.3028	0.7654	0.5511	0.029*	
H20C	1.0702	0.7745	0.5469	0.029*	
C31	1.7444 (6)	0.5077 (3)	1.0259 (3)	0.0093 (9)	
C32	1.7685 (6)	0.5861 (3)	0.9984 (3)	0.0109 (10)	
C33	1.7570 (6)	0.6482 (3)	1.0467 (3)	0.0123 (10)	
H33	1.7725	0.7005	1.0286	0.015*	
C34	1.7222 (7)	0.6355 (3)	1.1238 (3)	0.0158 (10)	
H34	1.7153	0.6794	1.1568	0.019*	
C35	1.6983 (7)	0.5611 (3)	1.1512 (3)	0.0142 (10)	
H35	1.6745	0.5536	1.2030	0.017*	
C36	1.7087 (6)	0.4953 (3)	1.1028 (3)	0.0131 (10)	
C37	1.6868 (7)	0.4162 (3)	1.1267 (3)	0.0139 (10)	
H37	1.6628	0.4054	1.1779	0.017*	
C38	1.6998 (7)	0.3554 (3)	1.0765 (3)	0.0130 (10)	
H38	1.6866	0.3026	1.0935	0.016*	
C39	1.7329 (6)	0.3701 (3)	0.9996 (3)	0.0108 (9)	
C40	1.7473 (7)	0.3053 (3)	0.9442 (3)	0.0150 (10)	
H40A	1.7911	0.3270	0.8968	0.022*	
H40B	1.6184	0.2785	0.9342	0.022*	
H40C	1.8420	0.2677	0.9650	0.022*	
C41	1.4473 (7)	1.2813 (3)	0.6720 (3)	0.0219 (12)	
H41A	1.5213	1.3054	0.6321	0.033*	
H41B	1.5322	1.2467	0.7027	0.033*	
H41C	1.4016	1.3228	0.7044	0.033*	
C42	1.9073 (7)	0.7824 (3)	0.8360 (3)	0.0195 (11)	
H42A	1.9553	0.8313	0.8621	0.029*	
H42B	1.8337	0.7950	0.7881	0.029*	
H42C	2.0189	0.7506	0.8255	0.029*	

### Atomic displacement parameters (Å^2^)

**Table:**

	*U*^11^	*U*^22^	*U*^33^	*U*^12^	*U*^13^	*U*^23^
Sn1	0.00955 (15)	0.00599 (17)	0.00853 (16)	0.00151 (11)	0.00198 (12)	0.00095 (12)
Sn2	0.01070 (15)	0.00607 (17)	0.00769 (16)	0.00190 (12)	0.00154 (12)	−0.00014 (12)
Cl1	0.0160 (5)	0.0070 (5)	0.0141 (6)	0.0036 (4)	0.0042 (4)	−0.0003 (4)
Cl2	0.0179 (6)	0.0132 (6)	0.0101 (6)	0.0026 (4)	0.0025 (4)	0.0033 (4)
Cl3	0.0113 (5)	0.0116 (6)	0.0140 (6)	−0.0030 (4)	0.0019 (4)	0.0006 (5)
Cl4	0.0114 (5)	0.0141 (6)	0.0220 (6)	−0.0019 (4)	0.0028 (5)	−0.0015 (5)
Cl5	0.0175 (6)	0.0092 (6)	0.0141 (6)	0.0045 (4)	−0.0019 (4)	0.0010 (4)
Cl6	0.0172 (5)	0.0124 (6)	0.0089 (5)	0.0023 (4)	0.0025 (4)	−0.0028 (4)
Cl7	0.0117 (5)	0.0130 (6)	0.0175 (6)	−0.0013 (4)	0.0022 (4)	0.0026 (5)
Cl8	0.0120 (5)	0.0139 (6)	0.0139 (6)	−0.0019 (4)	0.0015 (4)	−0.0010 (5)
O1	0.0144 (16)	0.0110 (17)	0.0087 (16)	0.0055 (13)	0.0004 (13)	0.0032 (13)
O2	0.0200 (18)	0.0168 (19)	0.0111 (17)	0.0054 (15)	0.0016 (14)	0.0061 (15)
O3	0.0176 (17)	0.0092 (17)	0.0087 (16)	0.0024 (13)	0.0009 (13)	−0.0034 (13)
O4	0.030 (2)	0.0126 (19)	0.0098 (17)	0.0036 (15)	0.0056 (15)	−0.0017 (14)
O5	0.0252 (19)	0.0099 (18)	0.0115 (17)	0.0035 (15)	0.0030 (15)	−0.0038 (14)
O6	0.0227 (18)	0.0116 (18)	0.0105 (17)	0.0022 (15)	0.0055 (14)	0.0020 (14)
O7	0.0184 (18)	0.015 (2)	0.034 (2)	0.0076 (15)	−0.0065 (17)	−0.0047 (17)
O8	0.024 (2)	0.0103 (18)	0.023 (2)	0.0060 (15)	0.0056 (16)	0.0068 (15)
N1	0.0115 (19)	0.0030 (19)	0.012 (2)	−0.0017 (15)	0.0021 (15)	−0.0001 (15)
N2	0.013 (2)	0.008 (2)	0.018 (2)	−0.0023 (16)	0.0019 (17)	−0.0015 (17)
N3	0.0077 (18)	0.0044 (19)	0.0121 (19)	0.0004 (14)	0.0002 (15)	0.0000 (15)
N4	0.0107 (19)	0.012 (2)	0.018 (2)	0.0024 (16)	−0.0006 (16)	−0.0004 (17)
N5	0.0082 (18)	0.010 (2)	0.011 (2)	0.0031 (15)	0.0021 (15)	−0.0022 (16)
N6	0.0080 (18)	0.009 (2)	0.011 (2)	0.0017 (15)	0.0006 (15)	0.0004 (16)
C1	0.010 (2)	0.004 (2)	0.013 (2)	−0.0035 (17)	−0.0013 (17)	−0.0001 (18)
C2	0.011 (2)	0.008 (2)	0.010 (2)	−0.0027 (18)	−0.0002 (18)	−0.0029 (18)
C3	0.014 (2)	0.011 (2)	0.016 (3)	0.0020 (19)	0.0033 (19)	0.001 (2)
C4	0.009 (2)	0.011 (2)	0.020 (3)	0.0015 (18)	0.0024 (19)	−0.007 (2)
C5	0.014 (2)	0.006 (2)	0.010 (2)	−0.0031 (17)	0.0034 (18)	−0.0021 (18)
C6	0.015 (2)	0.008 (2)	0.012 (2)	0.0008 (18)	0.0040 (18)	−0.0002 (18)
C7	0.011 (2)	0.006 (2)	0.011 (2)	−0.0035 (18)	0.0031 (18)	−0.0013 (18)
C8	0.014 (2)	0.011 (2)	0.016 (2)	0.0002 (18)	0.0056 (19)	−0.005 (2)
C9	0.012 (2)	0.012 (2)	0.014 (2)	0.0000 (18)	−0.0043 (18)	0.005 (2)
C10	0.014 (2)	0.016 (3)	0.010 (2)	0.0009 (19)	0.0021 (18)	−0.0031 (19)
C11	0.0080 (19)	0.007 (2)	0.014 (2)	0.0022 (17)	−0.0015 (18)	0.0032 (19)
C12	0.005 (2)	0.014 (2)	0.015 (2)	0.0025 (17)	0.0041 (18)	0.001 (2)
C13	0.014 (2)	0.012 (2)	0.014 (2)	0.0013 (19)	0.0013 (19)	0.001 (2)
C14	0.018 (2)	0.012 (2)	0.020 (3)	0.0015 (19)	−0.001 (2)	0.010 (2)
C15	0.018 (2)	0.021 (3)	0.014 (3)	0.000 (2)	0.003 (2)	0.005 (2)
C16	0.008 (2)	0.016 (3)	0.012 (2)	0.0032 (18)	0.0004 (18)	0.001 (2)
C17	0.021 (3)	0.014 (3)	0.007 (2)	0.006 (2)	−0.0059 (19)	−0.0059 (19)
C18	0.017 (2)	0.011 (2)	0.015 (2)	0.0006 (19)	−0.0013 (19)	−0.004 (2)
C19	0.007 (2)	0.011 (2)	0.020 (3)	0.0008 (18)	0.0016 (19)	−0.001 (2)
C20	0.023 (3)	0.017 (3)	0.018 (3)	−0.003 (2)	0.006 (2)	0.001 (2)
C31	0.006 (2)	0.007 (2)	0.015 (2)	0.0024 (17)	−0.0005 (18)	−0.0010 (19)
C32	0.007 (2)	0.014 (2)	0.013 (2)	0.0034 (18)	0.0024 (18)	0.0045 (19)
C33	0.012 (2)	0.009 (2)	0.016 (2)	0.0018 (18)	−0.0023 (19)	−0.0016 (19)
C34	0.013 (2)	0.017 (3)	0.018 (3)	0.005 (2)	0.001 (2)	0.000 (2)
C35	0.012 (2)	0.016 (3)	0.015 (2)	0.0011 (19)	0.003 (2)	−0.003 (2)
C36	0.010 (2)	0.014 (3)	0.016 (2)	0.0065 (18)	0.0002 (18)	0.002 (2)
C37	0.014 (2)	0.017 (3)	0.011 (2)	−0.001 (2)	0.0028 (19)	0.003 (2)
C38	0.013 (2)	0.010 (2)	0.017 (3)	−0.0024 (18)	0.0040 (19)	0.007 (2)
C39	0.009 (2)	0.009 (2)	0.015 (2)	0.0004 (18)	0.0016 (18)	−0.0017 (19)
C40	0.021 (3)	0.006 (2)	0.018 (3)	0.0001 (19)	0.001 (2)	−0.0026 (19)
C41	0.022 (3)	0.022 (3)	0.022 (3)	0.001 (2)	0.004 (2)	0.002 (2)
C42	0.018 (2)	0.022 (3)	0.019 (3)	0.000 (2)	0.003 (2)	0.003 (2)

### Geometric parameters (Å, °)

**Table:**

Sn1—O1	2.089 (3)	C8—H8	0.9500
Sn1—N1	2.265 (4)	C9—C10	1.393 (7)
Sn1—Cl1	2.3617 (11)	C9—H9	0.9500
Sn1—Cl4	2.3752 (11)	C10—H10	0.9500
Sn1—Cl2	2.3906 (12)	C11—C16	1.392 (6)
Sn1—Cl3	2.4089 (11)	C11—C12	1.416 (7)
Sn2—O3	2.096 (3)	C12—C13	1.368 (7)
Sn2—N3	2.275 (4)	C13—C14	1.410 (7)
Sn2—Cl5	2.3605 (12)	C13—H13	0.9500
Sn2—Cl6	2.3806 (11)	C14—C15	1.368 (7)
Sn2—Cl7	2.3862 (11)	C14—H14	0.9500
Sn2—Cl8	2.4079 (11)	C15—C16	1.409 (7)
O1—C1	1.308 (5)	C15—H15	0.9500
O2—C1	1.205 (6)	C16—C17	1.420 (7)
O3—C6	1.292 (6)	C17—C18	1.355 (7)
O4—C6	1.221 (6)	C17—H17	0.9500
O5—C12	1.347 (6)	C18—C19	1.401 (7)
O5—H5O	0.8400	C18—H18	0.9500
O6—C32	1.356 (6)	C19—C20	1.486 (7)
O6—H6O	0.8400	C20—H20A	0.9800
O7—C41	1.415 (6)	C20—H20B	0.9800
O7—H7O	0.8400	C20—H20C	0.9800
O8—C42	1.414 (6)	C31—C36	1.413 (7)
O8—H8O	0.8400	C31—C32	1.426 (6)
N1—C2	1.337 (6)	C32—C33	1.361 (7)
N1—C5	1.338 (6)	C33—C34	1.415 (7)
N2—C4	1.330 (6)	C33—H33	0.9500
N2—C3	1.342 (6)	C34—C35	1.363 (7)
N3—C10	1.327 (6)	C34—H34	0.9500
N3—C7	1.336 (6)	C35—C36	1.411 (7)
N4—C9	1.334 (6)	C35—H35	0.9500
N4—C8	1.346 (6)	C36—C37	1.415 (7)
N5—C19	1.341 (6)	C37—C38	1.367 (7)
N5—C11	1.387 (6)	C37—H37	0.9500
N5—H5N	0.8800	C38—C39	1.416 (6)
N6—C39	1.322 (6)	C38—H38	0.9500
N6—C31	1.372 (6)	C39—C40	1.478 (6)
N6—H6N	0.8800	C40—H40A	0.9800
C1—C2	1.515 (6)	C40—H40B	0.9800
C2—C3	1.384 (6)	C40—H40C	0.9800
C3—H3	0.9500	C41—H41A	0.9800
C4—C5	1.407 (6)	C41—H41B	0.9800
C4—H4	0.9500	C41—H41C	0.9800
C5—H5	0.9500	C42—H42A	0.9800
C6—C7	1.515 (6)	C42—H42B	0.9800
C7—C8	1.385 (6)	C42—H42C	0.9800
			
O1—Sn1—N1	75.54 (13)	C9—C10—H10	119.8
O1—Sn1—Cl1	91.69 (9)	N5—C11—C16	120.1 (4)
N1—Sn1—Cl1	167.23 (10)	N5—C11—C12	118.6 (4)
O1—Sn1—Cl4	89.55 (9)	C16—C11—C12	121.3 (4)
N1—Sn1—Cl4	84.94 (10)	O5—C12—C13	125.8 (5)
Cl1—Sn1—Cl4	95.50 (4)	O5—C12—C11	116.0 (4)
O1—Sn1—Cl2	167.42 (9)	C13—C12—C11	118.2 (4)
N1—Sn1—Cl2	91.93 (10)	C12—C13—C14	121.0 (5)
Cl1—Sn1—Cl2	100.83 (4)	C12—C13—H13	119.5
Cl4—Sn1—Cl2	90.47 (4)	C14—C13—H13	119.5
O1—Sn1—Cl3	86.50 (9)	C15—C14—C13	120.8 (5)
N1—Sn1—Cl3	85.21 (10)	C15—C14—H14	119.6
Cl1—Sn1—Cl3	93.76 (4)	C13—C14—H14	119.6
Cl4—Sn1—Cl3	170.03 (4)	C14—C15—C16	119.6 (5)
Cl2—Sn1—Cl3	91.41 (4)	C14—C15—H15	120.2
O3—Sn2—N3	75.79 (13)	C16—C15—H15	120.2
O3—Sn2—Cl5	93.46 (10)	C11—C16—C15	119.2 (5)
N3—Sn2—Cl5	169.25 (10)	C11—C16—C17	117.4 (4)
O3—Sn2—Cl6	166.10 (10)	C15—C16—C17	123.4 (5)
N3—Sn2—Cl6	90.32 (10)	C18—C17—C16	120.4 (4)
Cl5—Sn2—Cl6	100.43 (4)	C18—C17—H17	119.8
O3—Sn2—Cl7	88.23 (9)	C16—C17—H17	119.8
N3—Sn2—Cl7	85.53 (10)	C17—C18—C19	121.5 (5)
Cl5—Sn2—Cl7	94.30 (4)	C17—C18—H18	119.3
Cl6—Sn2—Cl7	91.27 (4)	C19—C18—H18	119.3
O3—Sn2—Cl8	86.55 (9)	N5—C19—C18	118.1 (5)
N3—Sn2—Cl8	85.19 (10)	N5—C19—C20	119.4 (5)
Cl5—Sn2—Cl8	94.26 (4)	C18—C19—C20	122.5 (5)
Cl6—Sn2—Cl8	91.82 (4)	C19—C20—H20A	109.5
Cl7—Sn2—Cl8	170.23 (4)	C19—C20—H20B	109.5
C1—O1—Sn1	120.4 (3)	H20A—C20—H20B	109.5
C6—O3—Sn2	119.9 (3)	C19—C20—H20C	109.5
C12—O5—H5O	109.5	H20A—C20—H20C	109.5
C32—O6—H6O	109.5	H20B—C20—H20C	109.5
C41—O7—H7O	109.5	N6—C31—C36	119.6 (4)
C42—O8—H8O	109.5	N6—C31—C32	120.5 (4)
C2—N1—C5	118.8 (4)	C36—C31—C32	119.9 (4)
C2—N1—Sn1	112.3 (3)	O6—C32—C33	126.4 (5)
C5—N1—Sn1	128.9 (3)	O6—C32—C31	114.2 (4)
C4—N2—C3	117.0 (4)	C33—C32—C31	119.5 (4)
C10—N3—C7	118.6 (4)	C32—C33—C34	120.5 (5)
C10—N3—Sn2	130.2 (3)	C32—C33—H33	119.8
C7—N3—Sn2	111.2 (3)	C34—C33—H33	119.8
C9—N4—C8	116.8 (4)	C35—C34—C33	121.0 (5)
C19—N5—C11	122.5 (4)	C35—C34—H34	119.5
C19—N5—H5N	118.8	C33—C34—H34	119.5
C11—N5—H5N	118.8	C34—C35—C36	120.1 (5)
C39—N6—C31	123.9 (4)	C34—C35—H35	119.9
C39—N6—H6N	118.1	C36—C35—H35	119.9
C31—N6—H6N	118.1	C35—C36—C31	119.0 (5)
O2—C1—O1	126.0 (4)	C35—C36—C37	123.7 (5)
O2—C1—C2	119.0 (4)	C31—C36—C37	117.2 (4)
O1—C1—C2	115.0 (4)	C38—C37—C36	120.4 (4)
N1—C2—C3	120.7 (4)	C38—C37—H37	119.8
N1—C2—C1	116.8 (4)	C36—C37—H37	119.8
C3—C2—C1	122.6 (4)	C37—C38—C39	120.9 (5)
N2—C3—C2	121.9 (5)	C37—C38—H38	119.6
N2—C3—H3	119.1	C39—C38—H38	119.6
C2—C3—H3	119.1	N6—C39—C38	118.0 (4)
N2—C4—C5	122.1 (5)	N6—C39—C40	120.0 (4)
N2—C4—H4	118.9	C38—C39—C40	121.9 (4)
C5—C4—H4	118.9	C39—C40—H40A	109.5
N1—C5—C4	119.6 (5)	C39—C40—H40B	109.5
N1—C5—H5	120.2	H40A—C40—H40B	109.5
C4—C5—H5	120.2	C39—C40—H40C	109.5
O4—C6—O3	126.4 (5)	H40A—C40—H40C	109.5
O4—C6—C7	118.0 (4)	H40B—C40—H40C	109.5
O3—C6—C7	115.6 (4)	O7—C41—H41A	109.5
N3—C7—C8	120.7 (4)	O7—C41—H41B	109.5
N3—C7—C6	117.4 (4)	H41A—C41—H41B	109.5
C8—C7—C6	121.9 (4)	O7—C41—H41C	109.5
N4—C8—C7	121.5 (4)	H41A—C41—H41C	109.5
N4—C8—H8	119.2	H41B—C41—H41C	109.5
C7—C8—H8	119.2	O8—C42—H42A	109.5
N4—C9—C10	121.9 (5)	O8—C42—H42B	109.5
N4—C9—H9	119.0	H42A—C42—H42B	109.5
C10—C9—H9	119.0	O8—C42—H42C	109.5
N3—C10—C9	120.4 (4)	H42A—C42—H42C	109.5
N3—C10—H10	119.8	H42B—C42—H42C	109.5
			
N1—Sn1—O1—C1	0.3 (3)	O4—C6—C7—C8	1.8 (7)
Cl1—Sn1—O1—C1	179.9 (3)	O3—C6—C7—C8	−178.8 (4)
Cl4—Sn1—O1—C1	−84.6 (3)	C9—N4—C8—C7	0.7 (7)
Cl2—Sn1—O1—C1	5.5 (7)	N3—C7—C8—N4	0.3 (7)
Cl3—Sn1—O1—C1	86.3 (3)	C6—C7—C8—N4	−178.6 (4)
N3—Sn2—O3—C6	−2.2 (3)	C8—N4—C9—C10	−0.6 (7)
Cl5—Sn2—O3—C6	177.8 (3)	C7—N3—C10—C9	1.5 (7)
Cl6—Sn2—O3—C6	−4.5 (6)	Sn2—N3—C10—C9	−176.7 (3)
Cl7—Sn2—O3—C6	83.6 (3)	N4—C9—C10—N3	−0.5 (7)
Cl8—Sn2—O3—C6	−88.2 (3)	C19—N5—C11—C16	0.8 (6)
O1—Sn1—N1—C2	−1.4 (3)	C19—N5—C11—C12	180.0 (4)
Cl1—Sn1—N1—C2	−3.1 (7)	N5—C11—C12—O5	1.1 (6)
Cl4—Sn1—N1—C2	89.5 (3)	C16—C11—C12—O5	−179.7 (4)
Cl2—Sn1—N1—C2	179.8 (3)	N5—C11—C12—C13	−179.6 (4)
Cl3—Sn1—N1—C2	−89.0 (3)	C16—C11—C12—C13	−0.4 (7)
O1—Sn1—N1—C5	179.1 (4)	O5—C12—C13—C14	−179.8 (4)
Cl1—Sn1—N1—C5	177.3 (3)	C11—C12—C13—C14	1.0 (7)
Cl4—Sn1—N1—C5	−90.1 (4)	C12—C13—C14—C15	−1.5 (7)
Cl2—Sn1—N1—C5	0.2 (4)	C13—C14—C15—C16	1.3 (7)
Cl3—Sn1—N1—C5	91.5 (4)	N5—C11—C16—C15	179.5 (4)
O3—Sn2—N3—C10	−178.4 (4)	C12—C11—C16—C15	0.3 (7)
Cl5—Sn2—N3—C10	−178.3 (4)	N5—C11—C16—C17	−1.2 (6)
Cl6—Sn2—N3—C10	1.0 (4)	C12—C11—C16—C17	179.6 (4)
Cl7—Sn2—N3—C10	92.3 (4)	C14—C15—C16—C11	−0.7 (7)
Cl8—Sn2—N3—C10	−90.8 (4)	C14—C15—C16—C17	179.9 (5)
O3—Sn2—N3—C7	3.3 (3)	C11—C16—C17—C18	1.0 (7)
Cl5—Sn2—N3—C7	3.4 (7)	C15—C16—C17—C18	−179.6 (5)
Cl6—Sn2—N3—C7	−177.3 (3)	C16—C17—C18—C19	−0.4 (7)
Cl7—Sn2—N3—C7	−86.0 (3)	C11—N5—C19—C18	−0.2 (6)
Cl8—Sn2—N3—C7	90.9 (3)	C11—N5—C19—C20	179.3 (4)
Sn1—O1—C1—O2	−178.2 (4)	C17—C18—C19—N5	0.0 (7)
Sn1—O1—C1—C2	0.7 (5)	C17—C18—C19—C20	−179.5 (5)
C5—N1—C2—C3	0.8 (7)	C39—N6—C31—C36	−1.1 (7)
Sn1—N1—C2—C3	−178.7 (3)	C39—N6—C31—C32	179.7 (4)
C5—N1—C2—C1	−178.2 (4)	N6—C31—C32—O6	−1.9 (6)
Sn1—N1—C2—C1	2.2 (5)	C36—C31—C32—O6	178.9 (4)
O2—C1—C2—N1	177.0 (4)	N6—C31—C32—C33	179.3 (4)
O1—C1—C2—N1	−2.0 (6)	C36—C31—C32—C33	0.1 (7)
O2—C1—C2—C3	−2.1 (7)	O6—C32—C33—C34	−178.5 (4)
O1—C1—C2—C3	178.9 (4)	C31—C32—C33—C34	0.2 (7)
C4—N2—C3—C2	0.6 (7)	C32—C33—C34—C35	−0.4 (7)
N1—C2—C3—N2	−0.3 (7)	C33—C34—C35—C36	0.2 (7)
C1—C2—C3—N2	178.8 (4)	C34—C35—C36—C31	0.1 (7)
C3—N2—C4—C5	−1.5 (7)	C34—C35—C36—C37	179.6 (4)
C2—N1—C5—C4	−1.7 (6)	N6—C31—C36—C35	−179.4 (4)
Sn1—N1—C5—C4	177.8 (3)	C32—C31—C36—C35	−0.2 (7)
N2—C4—C5—N1	2.1 (7)	N6—C31—C36—C37	1.0 (6)
Sn2—O3—C6—O4	−179.7 (4)	C32—C31—C36—C37	−179.8 (4)
Sn2—O3—C6—C7	0.9 (5)	C35—C36—C37—C38	−179.6 (4)
C10—N3—C7—C8	−1.4 (6)	C31—C36—C37—C38	−0.1 (7)
Sn2—N3—C7—C8	177.1 (3)	C36—C37—C38—C39	−0.8 (7)
C10—N3—C7—C6	177.6 (4)	C31—N6—C39—C38	0.2 (6)
Sn2—N3—C7—C6	−3.9 (5)	C31—N6—C39—C40	−179.1 (4)
O4—C6—C7—N3	−177.1 (4)	C37—C38—C39—N6	0.8 (7)
O3—C6—C7—N3	2.3 (6)	C37—C38—C39—C40	−179.9 (4)

### Hydrogen-bond geometry (Å, °)

**Table:**

*D*—H···*A*	*D*—H	H···*A*	*D*···*A*	*D*—H···*A*
O5—H5o···O7	0.84	1.84	2.661 (5)	167
O7—H7o···N2	0.84	2.05	2.871 (5)	165
O6—H6o···O8	0.84	1.82	2.649 (5)	171
O8—H8o···N4	0.84	2.10	2.924 (5)	168
N5—H5n···O4	0.88	1.88	2.731 (5)	163
N6—H6n···O2^i^	0.88	1.98	2.838 (5)	164

Symmetry codes: (i) *x*+1, *y*−1, *z*.
